# Selection and evaluation of DNase I hypersensitive sites for prenatal screening of trisomy 21 in the fetus

**DOI:** 10.18699/vjgb-26-67

**Published:** 2026-07

**Authors:** A.M. Mazur, A.S. Starshin, N.V. Bogush, E.B. Prokhortchouk

**Affiliations:** Institute of Bioengineering, Research Center of Biotechnology of the Russian Academy of Sciences, Moscow, Russia; Institute of Bioengineering, Research Center of Biotechnology of the Russian Academy of Sciences, Moscow, Russia; N.I. Pirogov Russian National Research Medical University, Moscow, Russia; Institute of Bioengineering, Research Center of Biotechnology of the Russian Academy of Sciences, Moscow, Russia Sirius University of Science and Technology, Sirius Federal Territory, Krasnodar region, Russia

**Keywords:** chromatin, cell free DNA, aneuploidy, trisomy, NIPT, хроматин, внеклеточная ДНК, анеуплоидия, трисомия, НИПТ

## Abstract

This study introduces a novel approach for noninvasive prenatal testing (NIPT) of chromosomal abnormalities, based on analysis of epigenetic features in circulating cell-free DNA (cfDNA). The core innovation of our method leverages fundamental differences in chromatin organization between maternal and fetal cells. Specifically, we focused on genomic regions that exhibit open chromatin configuration in maternal blood cells but remain tightly packed in fetal tissues (DNase I hypersensitive sites or DHSs). These epigenetic differences create distinct cfDNA fragmentation signatures that allow selective identification of fetal DNA within the maternal cfDNA pool. The study workflow comprised several key steps: performing genome-wide screening to identify differentially accessible chromatin regions, selecting the most informative markers using a machine learning algorithm, and targeted sequencing of the selected epigenetic markers using molecular barcodes. Subsequently, a LASSO regression model was constructed and validated. As a proof of concept, the study demonstrates the method’s efficacy in identifying trisomy 21 (Down syndrome), though the underlying principles can be readily adapted to other abnormalities. Complementing its robust performance, the technique offers practical advantages in terms of platform compatibility – the same epigenetic markers can be assessed using either next-generation sequencing or simpler, more cost-efficient methods like digital PCR. With further refinement, the approach could be extended to screen for additional aneuploidies (trisomies 13 and 18) and microdeletion syndromes. Therefore, this approach offers new opportunities for developing cost-effective testing systems suitable for widespread routine clinical implementation, combining high diagnostic accuracy with reduced analysis costs.

## Abstract (ru)

В данной работе представлен новый подход к неинвазивной пренатальной диагностике хромосомных аномалий, основанный на анализе эпигенетических особенностей циркулирующей внеклеточной ДНК (вкДНК). Ключевая идея метода заключается в использовании фундаментальных различий в организации хроматина между материнскими и фетальными клетками. В частности, мы сосредоточились на участках генома, которые в материнских клетках крови находятся в открытом состоянии (DHS), но остаются закрытыми в тканях плода. Эти эпигенетические различия создают уникальные паттерны вкДНК, позволяющие выделить фетальную ДНК из общего пула материнской вкДНК. Работа включает в себя несколько основных шагов: проведение полногеномного скрининга для выявления дифференциально доступных участков хроматина, отбор наиболее информативных маркеров при помощи алгоритма машинного обучения, таргетное секвенирование отобранных эпигенетических маркеров с применением молекулярных баркодов и построение модели на основе LASSO-регрессии с последующей ее валидацией. Особое внимание уделено диагностике трисомии хромосомы 21 (синдрома Дауна), как наиболее клинически значимого хромосомного нарушения. Разработанный алгоритм демонстрирует высокую точность при анализе отобранных эпигенетических маркеров. Важное преимущество метода – его технологическая гибкость: отобранные маркеры можно анализировать как с помощью NGS, так и более доступными методами, включая цифровую ПЦР. Перспективы дальнейшего развития метода включают расширение диагностической панели для выявления других частых анеуплоидий (трисомии хромосом 13, 18), а также микроделеционных синдромов. Предлагаемый подход открывает новые возможности для создания экономически эффективных тест-систем, которые могут быть внедрены в широкую клиническую практику, обеспечивая высокую точность диагностики при снижении стоимости анализа.

## Introduction

The Non-Invasive Prenatal Test (NIPT), which employs next-generation sequencing (NGS) technology, represents a revolutionary approach to detecting fetal chromosomal abnormalities using only a sample of maternal blood (Fan et al., 2008). Prior to the development of NGS, this type of analysis was not feasible, as there was no reliable method to isolate the small amount of fetal DNA present amidst the vast excess of maternal DNA in the bloodstream (Dhallan et al., 2007). Due to the high throughput and sensitivity of NGS, it is now possible to accurately detect and quantify these trace amounts of fetal cell-free DNA (cfDNA) directly from maternal plasma (Alberry et al., 2021). Consequently, NIPT has emerged as a rapid, safe, and reliable screening tool for assessing the risk of common aneuploidies, including Down, Edwards, and Patau syndromes (Gil et al., 2017). The genomics revolution driven by NGS has therefore paved the way for the widespread clinical implementation of NIPT (Satam et al., 2023).

However, despite the impressive capabilities of NGS, the effectiveness of NIPT still depends on selecting optimal biological markers to distinguish between maternal and fetal DNA (Hui, Bianchi, 2020). In this context, epigenetic differences – particularly chromatin accessibility profiles – are of special interest.

DNase I-hypersensitive sites (DHSs) are genomic regions with an open chromatin structure, exhibiting increased accessibility to the DNase I enzyme. These sites are frequently associated with gene regulatory elements and display cellspecific expression profiles (Thurman et al., 2012).

In the previous stage of this study, the use of DHS clusters significantly restricted the number of available candidate regions, thereby reducing the sensitivity and specificity of the test system. To overcome this limitation, we transitioned from cluster analysis to the investigation of individual DHS. This approach substantially expanded the pool of candidate regions, enabling more precise filtering and ultimately yielding a sufficient number of high-quality loci for reliable diagnostics.

Our study focuses on developing an enhanced NIPT approach based on the analysis of individual DHSs – genomic regions with chromatin structures characteristic of the fetus and mother. The proposed method is expected to ensure high accuracy in fetal DNA detection by leveraging the epigenetic features of DHSs, while also reducing assay costs through their targeted analysis

## Materials and methods

Study cohort. A total of 1,012 blood samples from pregnant women were collected between April 2014 and April 2015. The patients were referred from 47 medical institutions across the Russian Federation or were self-referred. The mean age of participants was 35 years, with the youngest being 20 and the oldest 51 years. Gestational age at the time of blood collection ranged from 10 to 24 weeks, with a mean of 14 weeks. The mean fetal cell-free DNA (cfDNA) concentration was 11 % (Pantiuhk et al., 2016). All women provided informed consent for the use of their samples for scientific purposes. The study was approved by the Committee of the Academic Council of the Faculty of Biomedical Sciences, Pirogov Russian National Research Medical University, Ministry of Health of the Russian Federation (protocol No. 4 dated December 26, 2022).

Primer design. Primers were designed using the Primer3 software (Untergasser et al., 2012) with the following parameters: PRIMER_OPT_SIZE = 21, PRIMER_MIN_SIZE = 18, PRIMER_MAX_SIZE = 24, PRIMER_MAX_NS_ACCEPTED = 1, and PRIMER_PRODUCT_SIZE_RANGE = 100–140 bp

Target enrichment and library preparation. Targeted sequencing was performed using single-end enrichment with duplex unique molecular identifiers (UMIs), following the protocol described by Q. Peng et al. (2019).

Library sequencing. Sequencing was carried out using Illumina SBS technology with single-end reads of at least 80 bp.

Model development and evaluation. In the first step, the input data were prepared by dividing samples into two categories: those with trisomy and those without it. Samples with a total molecule count below 15,000 were filtered out.

Subsequently, 100 independent trials were conducted using the R packages rsample and caret (Kuhn, 2008). In each trial, the data were randomly split into training (60 % of samples),

validation (20 %), and test (20 %) sets, while preserving the original class ratio.

To compensate for class imbalance in the training sets, observation weighting was applied. Class weights were calculated separately for each training set according to the formulas:

**Formula. 1.**
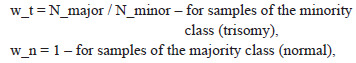
Formula 1

where N_major and N_minor are the number of samples in the majority and minority classes, respectively, within the training set. This weighting effectively increases the contribution of rare trisomy cases to the loss function, preventing the model from biasing toward the more common class.

The optimal classification threshold was determined on the validation set using Youden’s index (Youden, 1950), which maximizes the J statistic (J = sensitivity + specificity – 1). This criterion aims to find a threshold that balances the method’s ability to correctly identify both positive cases (sensitivity) and negative cases (specificity). This approach ensures that the selected threshold balances the two types of errors: false negatives (where aneuploidy is present but the test fails to detect it) and false positives (where the test incorrectly indicates aneuploidy). Three key performance metrics were calculated on the test data: the area under the ROC curve (AUC), sensitivity, and specificity. Model training was performed using the glmnet package (Friedman et al., 2010), and all results were visualized using ggplot2 (Wickham, 2016) and ggpubr.

## Results

**Identification of candidate regions in the human genome**

At the first stage, DNase I-hypersensitive sites (DHSs) were selected from an open-access database (Sheffield et al., 2013). For maternal tissue representation, all hematopoietic and endothelial cell lines were used, as these are typical sources of freely circulating maternal cfDNA (Lui et al., 2002).

The selection criterion was based on differential accessibility of these sites: an open chromatin state in cell lines corresponding to maternal tissues (31 lines of hematopoietic and endothelial origin) and a closed state in fetal cell lines (Chorion). This approach enabled the identification of 13,637 individual DHSs (Fig. S1)1.

Supplementary Materials are available in the online version of the paper: https://vavilov.elpub.ru/jour/manager/files/Suppl_Mazur_Engl_30_4.xlsx


The coordinates of all selected sites were converted to the human genome version GRCh38 (Table S1). For subsequent analysis, a 1,000 bp region was extracted around each 150 bp DHS, with the DHS positioned at its center. Although the DHSs themselves did not overlap, the flanking regions could partially overlap when the distance between central sites was less than 500 bp – a factor that was taken into account during final selection.

To construct the diagnostic panel, we selected a set of regions containing DHSs on: chromosome 21 – as the direct target for Down syndrome diagnosis; chromosome Y – as a negative control; and chromosomes 1, 2, 3, 4, 5, 6, 8, 10, and 12 – as markers, the relative coverage decrease of which in trisomy cases reflects the redistribution of reads in favor of chromosome 21.

**Sample set formation**

The study utilized cfDNA sequencing data from a previous work focused on non-invasive prenatal diagnostic methods for aneuploidies (Pantiukh et al., 2016).

To determine fetal cfDNA concentration, two complementary methods were applied: quantitative analysis of the Y chromosome and the SeqFF method, which is based on regression analysis of overrepresented fetal sequences (Kim et al., 2015; van Beek et al., 2017).

The need to group samples arose from the characteristics of the original data, where the mean coverage was only 0.3×. At this coverage level, a standard 150 bp DHS received on average only one read, which made reliable quantitative analysis at the individual level impossible. To address this low coverage issue, samples were sorted by increasing fetal DNA concentration and evenly distributed into four groups (P1–P4) (Fig. S2).

The resulting clinical sample set included data from 958 cases with normal fetal karyotype and 54 samples with trisomy 21 (Down syndrome).

**Whole-genome sequencing data analysis algorithm**

All selected cfDNA samples were aligned to the human reference genome GRCh38 using bowtie2 (Langmead, Salzberg, 2012) in global alignment mode (--end-to-end) with the --sensitive parameter. The mean percentage of successfully mapped reads was 97.05 %, with unmapped reads excluded from further analysis. For each sample, coverage was calculated for every nucleotide within the pre-selected regions

**Fig. 1.**
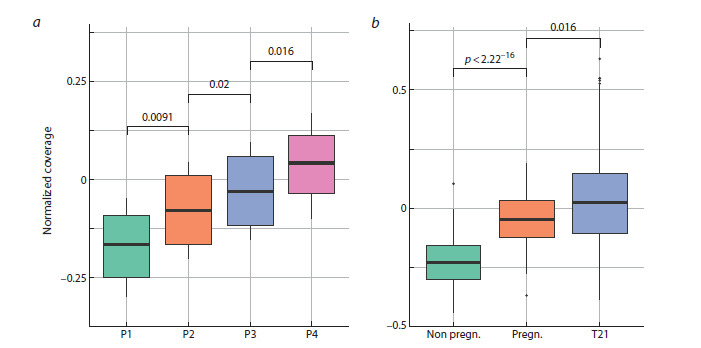
Normalized coverage (Z-score) in target regions using chromosome 21 as an example: a – in subgroups of pregnant women with normal fetal karyotype; b – in groups of non-pregnant women, pregnant women with normal fetal karyotype, and pregnant women with fetal trisomy. Statistical significance of differences between the groups was assessed using the Mann–Whitney U-test (Wilcoxon test for independent samples).

Aggregated data were divided into groups: non-pregnant women, pregnant women with normal fetal karyotype (further subdivided by fetal DNA concentration), and pregnant women with fetal trisomies. A statistically significant increase in coverage within DHSs was observed between groups of pregnant women with a normal fetal karyotype (Fig. 1a) and between pregnant women compared to non-pregnant women – the latter explained by the closed chromatin state of fetal DNA in these genomic regions, rendering it inaccessible to DNase I (Fig. 1b). Differences were also noted for pregnant women carrying fetuses with trisomy 21 (Fig. 1b). This increase in DHS coverage confirmed that the proposed method enables the detection of fetal aneuploidies.

Interestingly, multiple regions with increased coverage were identified in pregnant women. Verification using the UCSC Genome Browser confirmed the presence of additional DHSs within such regions (Fig. S3), which is consistent with known data on the clustering of regulatory elements in the genome (Thurman et al., 2012).

**Establishing criteria and selecting regions**

The primary objective was to identify regions most effective at distinguishing between pregnant and non-pregnant women. To this end, the following criteria were proposed: the length of the maximum window where the difference between mean coverage in non-pregnant women and mean coverage in pregnant women is less than zero; the maximum area under the difference curve within a 100-nucleotide window; the relationship between coverage level and fetal cfDNA concentration; and functional significance. Functional significance was assessed based on criteria proposed in R.E. Thurman et al. (2012). Promoter sites were defined as those the coordinates of which intersected with transcription start sites from the GENCODE human genome reference annotation (Harrow et al., 2012), or as the DHS nearest to a transcription start site in the 5ʹ direction. Enhancers were defined as those located within 500,000 nucleotides of a promoter site and showing high correlation (R ≥ 0.7) with that promoter site based on DNase-seq data.

To determine which of the proposed criteria was most suitable for region selection, a binary classifier based on random forest was constructed to distinguish samples from pregnant women versus non-pregnant women, using the mean coverage in the 20 top-ranking regions according to each criterion. We used the Random Forest Classifier algorithm from the scikitlearn library in its default configuration (Pedregosa et al., 2011). Prediction quality was assessed using the F-measure for non-pregnant sample predictions, as the number of such samples was smaller than that of pregnant samples. To identify the most effective region selection rule, we evaluated the predictive performance of each criterion individually and in various combinations. The best F-measure was achieved using regions selected by a combination of three criteria: the area under the difference curve criterion, the presence of a relationship between coverage level and fetal DNA concentration within the region, and the criterion of whether the site was a promoter or enhancer.

Next, for each nucleotide coordinate, the mean normalized coverage (Z-score) was calculated across all classes: for non-pregnant samples; for pregnant samples (by groups with different cfDNA concentrations); and for samples from pregnant women carrying fetuses with trisomy 21. Subsequently, all regions on a given chromosome were sorted in descending order according to the value determined by the selected criterion, and a specified number of regions were selected.

**Sequencing of the selected regions**

For the analysis of the 40 selected genomic regions (Table S2) across 283 samples, targeted sequencing based on multiplex PCR was employed. Specific primers were designed for each region (Table S3), enabling simultaneous amplification of all target sites in a single reaction. Prior to sequencing, molecular barcoding of the original DNA molecules was performed during the amplification step, ensuring accurate counting of initial molecule numbers and minimizing the impact of PCR artifacts. Read mapping was conducted in the same manner as described in the “Whole-genome sequencing data analysis algorithm” section.

**Model development and validation**

For 201 samples with a normal fetal karyotype and 82 samples with trisomy, counts of cfDNA molecules corresponding to the 40 selected regions were obtained. To reliably assess the suitability of these regions for use in a test system, we trained and tested a LASSO regression model using stratified repeated random subsampling with class weighting (see Material and methods).

**Fig. 2.**
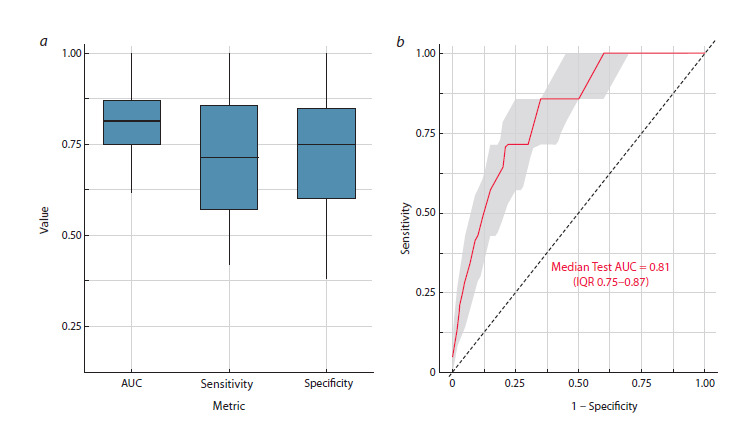
Model performance: a – distribution of metrics (AUC, sensitivity, and specificity); b – ROC curve based on 100 independent splits.

The high sensitivity and specificity values (Fig. 2a), along with the AUC (Fig. 2b) achieved by the model, allow us to conclude that it is possible to predict the presence of fetal trisomy based on sequencing data from DHS regions of circulating cfDNA.

## Discussion

Before the analysis of plasma cfDNA became the dominant approach in non-invasive prenatal testing, the scientific community was actively searching for alternative methods to obtain fetal genetic material without invasive procedures (Simpson, Elias, 1993).

Two main cell types served as primary targets in these studies: trophoblasts (Bruch et al., 1991) and nucleated red blood cells (fetal erythroblasts) (Simpson, Elias, 1994). Trophoblasts, being placental derivatives, seemed a logical target as they contain the complete fetal genome and could theoretically serve as ideal material for analysis (Covone et al., 1984). Concurrently, research was conducted on isolating fetal erythroblasts (Bianchi et al., 1990). The advantage of this approach lay in the fact that immature nucleated red blood cells are normally virtually absent from the peripheral blood of healthy adults, theoretically allowing them to be distinguished against the background of maternal cells.

However, practical application of the cellular approach encountered serious limitations (Holzgreve et al., 1992). The main problem was the extremely low concentration of fetal cells in maternal blood, making their isolation technically challenging and unreliable.Against this backdrop, the discovery made by Dennis Lo and colleagues in 1997 – demonstrating the presence of significant amounts of fetal cfDNA in maternal plasma – marked the beginning of a fundamentally different path (Lo et al., 1997). Despite the fact that cfDNA represented fragmented placental DNA rather than the complete fetal genome, this approach had decisive practical advantages: a relatively high concentration of genetic material and technological accessibility for analysis using PCR (Lo et al., 1998).

Current commercial NIPTs utilizing high-throughput sequencing methods demonstrate high accuracy, but their widespread adoption is still constrained by relatively high costs (Gil et al., 2017).

Our method, based on targeted analysis of 40 epigenetic markers (DHSs), offers a cost-effective solution. Such approaches require sequencing less than 1 % of the genome, substantially reducing reagent and data processing costs (Schmitt et al., 2015). It is worth noting that not only NGS platforms but also the more accessible digital PCR (dPCR) technology can be used for this analysis, further lowering costs and simplifying implementation into routine clinical practice (Li et al., 2022). This is particularly relevant for smaller laboratories without access to expensive sequencing systems. At the same time, all key advantages of NIPT are preserved: non-invasiveness, the possibility of early diagnosis, and high accuracy in detecting chromosomal abnormalities (Zhang et al., 2026).

An important aspect of this study was the development of rigorous criteria for selecting informative regions. Our method incorporates a comprehensive set of parameters, including not only statistical measures of differential accessibility but also the functional significance of sites and the dependence of the signal on fetal DNA concentration. Such an integrated approach enables the creation of a robust diagnostic panel resilient to technical noise. Furthermore, the application of molecular barcoding addresses a crucial challenge in quantitative cfDNA analysis – accurate counting of original molecules in the presence of PCR amplification.

## Conclusion

The prospects for practical application of the developed approach appear promising. First, the method fundamentally allows for expanding the range of detectable chromosomal abnormalities without substantially increasing analysis costs. Second, its adaptability to various technological platforms opens up possibilities for creating flexible diagnostic systems that can be optimized for specific clinical tasks and budget constraints.

Future research may focus on optimizing the marker panel for simultaneous detection of a wide range of chromosomal abnormalities and standardizing protocols for use in routine clinical practice. The proposed method has every reason to become the foundation for a new generation of screening tests that combine high reliability with cost-effectiveness.

## Conflict of interest

The authors declare no conflict of interest.
